# Microsnare Retrieval of a Distorted Flow Re-direction Endoluminal Device (FRED)

**DOI:** 10.7759/cureus.19803

**Published:** 2021-11-22

**Authors:** Ryan Johnson, Michael Young, Hamad Farhat

**Affiliations:** 1 Neurosurgery, Carle BroMenn Medical Center, Normal, USA; 2 Neurological Surgery, Advocate Christ Medical Center, Oak Lawn, USA

**Keywords:** intracranial aneurysm, flow diversion, snare, stent, flow re-direction endoluminal device

## Abstract

Retained endovascular devices are becoming increasingly reported as the indications for endovascular intervention continue to expand. As such, an interventionalist needs to be prepared to extract devices that are improperly deployed. This case illustrates the successful retrieval of an incompletely opened flow diverting stent using a microsnare. This is the second reported case of this complication and the first known case specific to the flow re-direction endoluminal device (FRED; Microvention, Aliso Viejo, California, USA).

## Introduction

Flow diversion has emerged as a workhorse of intracranial aneurysm treatment, especially for large, wide-necked, unruptured intracranial aneurysms. Multiple randomized, prospective trials have demonstrated safety, efficacy, and ease of use of flow-diverting stents [1-3]. FRED, flow re-direction endoluminal device (Microvention, Aliso Viejo, California, USA), is one such flow diverting device that is FDA approved for endovascular treatment of wide-necked intracranial aneurysms arising from the petrous segment of the internal carotid artery (ICA) to ICA terminus with parent vessel diameter ≥ 2.0 mm and ≤ 5.0 mm [3].

The pivotal EuFRED (defined in appendix) and SAFE (defined in appendix) trials showed a complete aneurysm occlusion rate of 91.3% and 73.3%, respectively, at one-year follow-up and permanent morbidity and mortality rates of <2% and <3% for unruptured and recanalized aneurysms located in the anterior circulation [2-3]. Although safety and efficacy of the FRED were shown in these pivotal trials, several device-related events were reported, including detachment in the microcatheter hub, torquing of the stent, foreshortening, and incomplete opening upon stent deployment [2]. We report a case of successful retrieval of an incompletely opened flow diverting stent using a microsnare. This is the second reported case of this complication and the first known case specific to the flow re-direction endoluminal device (FRED).

## Case presentation

We present a case of a 72-year-old female with a known 4mm x 3mm left clinoidal internal carotid artery (ICA) aneurysm diagnosed six years prior to presentation presenting with worsening headaches for the last four days. CT and CTA (computed tomography angiography) of the head did not demonstrate any evidence of subarachnoid hemorrhage but did demonstrate an interval size increase, compared to a surveillance CTA three months prior, of the unruptured left clinoidal ICA aneurysm, now measuring 5.3mm x 3.7mm with bilobed irregular morphology. The patient’s neurologic examination was unremarkable for focal deficits. Her prior headache history was significant for migraines without aura; however, the patient indicated that her current headache was different in location and intensity, now located over the left hemicranium. Endovascular intervention was recommended based on the patient's new symptoms, as well as the increasing size of this left clinoidal ICA aneurysm with irregular morphology. Informal verbal and written consent were obtained from the patient to proceed with a flow re-direction endoluminal device (FRED; Microvention, Aliso Viejo, California, USA) in an attempt to obliterate the aneurysm through flow diversion. The patient was started on ticagrelor 90mg twice daily one day before the procedure due to an aspirin allergy.

**Figure 1:**
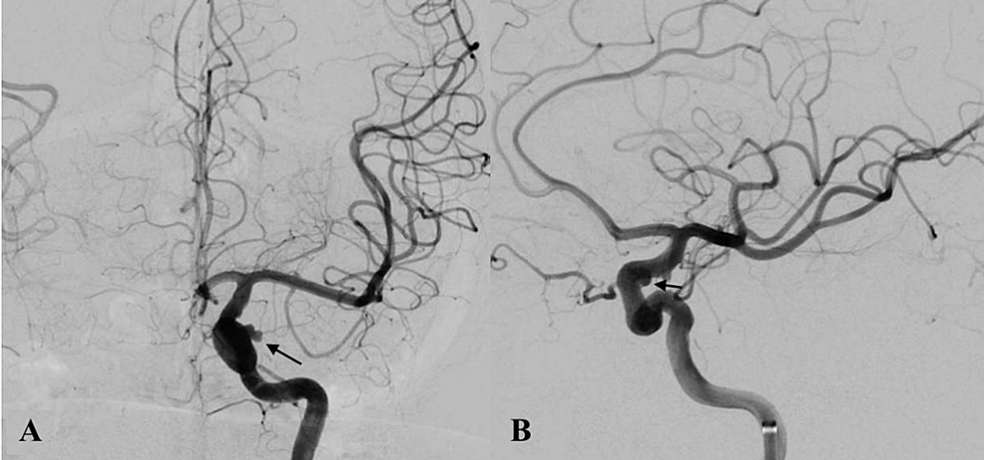
Anteroposterior (A) and lateral (B) internal carotid artery angiogram demonstrating a left clinoidal aneurysm (black arrow) with irregular morphology

The patient was brought to the neurointerventional suite, and the procedure was performed under general anesthesia. An 8F Cerebase guide sheath (Cerenovus, Miami, Florida, USA) was placed within the right femoral artery, and a 5F Berenstein diagnostic catheter (Cordis, Miami Lakes, Florida, USA) was advanced within the sheath to perform a left ICA angiogram showing the 5.3mm x 3.7mm left clinoidal ICA aneurysm with irregular morphology (Figure 1A-1B).

**Figure 2:**
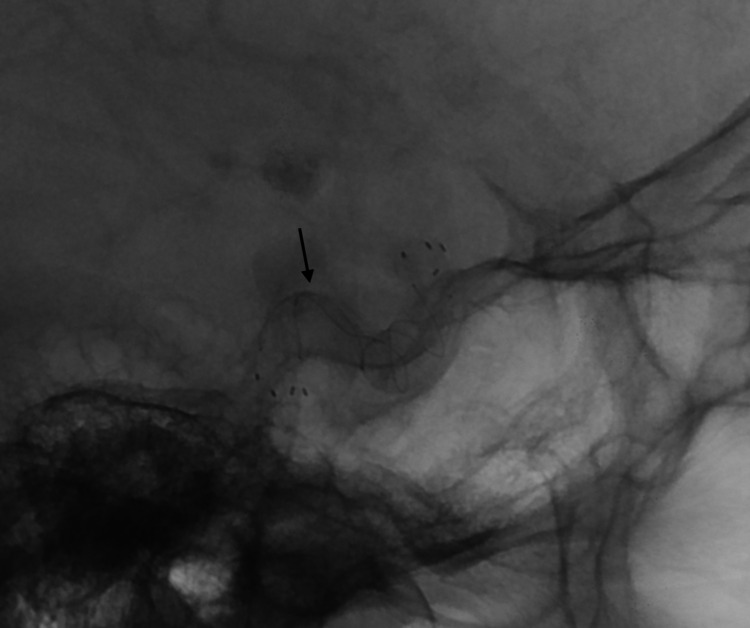
Lateral fluoroscopic image demonstrating distortion of the proximal portion of the flow diverting device. The black arrow demonstrates the portion of the device that incompletely opened.

The 8F Cerebase was then advanced into the proximal cavernous segment of the left ICA to provide proximal support. A Headway-27 microcatheter (Microvention, Aliso Viejo, California, USA) was inserted in the 8F Cerebase, and a Synchro-2 microwire (Stryker, Kalamazoo, Michigan, USA) was inserted in the microcatheter, and together were advanced into the distal left M1 segment. The microwire was removed, and a 5.5mm x 22mm FRED was advanced inside the microcatheter. The device was deployed with the distal anchors in the distal left ICA with adequate coverage of the left clinoidal aneurysm. After deployment, it was noticed that the device foreshortened and was not providing adequate coverage across the neck of the aneurysm. The decision was made to place another FRED to completely cover the aneurysm. Again, the Headway-27 microcatheter along with a Synchro-2 microwire was navigated into the left cavernous ICA. At this time, it was noticed the microwire was unable to navigate beyond the proximal segment of the device. Further, inspection revealed the FRED did not completely open and, instead, appeared to be distorted in the left cavernous ICA (Figure 2).

The decision was made to attempt the retrieval of the device using a microsnare. A 4mm loop-diameter x 200cm length Amplatz Goose Neck Microsnare (Medtronic, Irvine, California, USA) was inserted into the microcatheter to engage the proximal anchors of the device (Video 1). The device was successfully retrieved with the snare and pulled into the guide sheath. This process was repeated twice due to the dislodgement of the device from the snare during retrieval (Video 2).

**Video 1:** Lateral fluoroscopic projection of the distorted FRED demonstrating microsnare retrieval into the guide sheath.

**Video 2:** Anteroposterior fluoroscopic projection demonstrating microsnare retrieval of the FRED into the guide sheath.

**Figure 3:**
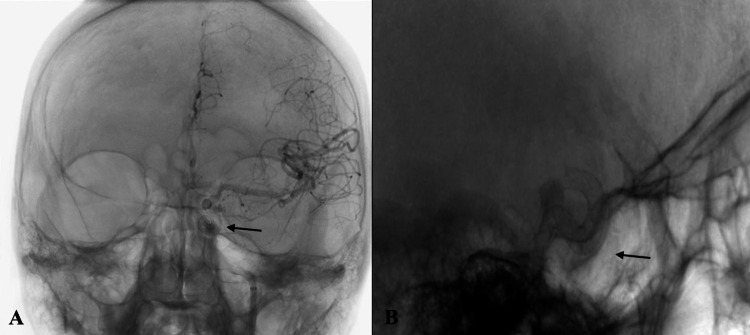
Anteroposterior (A) and lateral (B) internal carotid angiogram demonstrating proper placement of the PED (black arrow) over the neck of the left clinoidal aneurysm.

**Figure 4:**
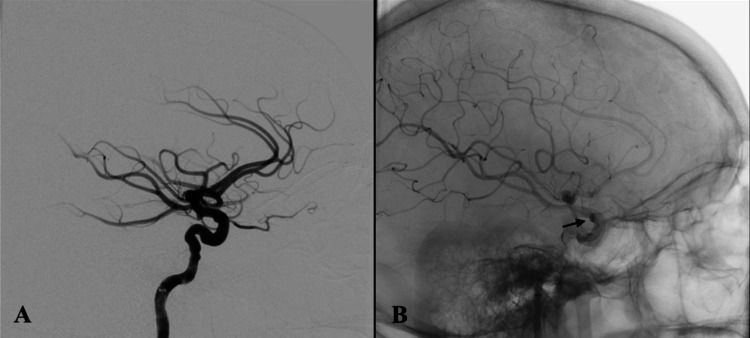
Post-pipeline deployment lateral internal carotid artery angiogram (A) demonstrating contrast stasis (black arrow) within the dome of the left clinoidal aneurysm (B).

Post-retrieval left ICA angiogram did not demonstrate any evidence of vessel injury. At this time, the decision was made to proceed with aneurysm treatment using a Pipeline Embolization Device (PED; Medtronic, Irvine, California, USA). A Phenom-27 microcatheter (Medtronic, Irvine, California, USA) along with a Synchro-2 microwire was inserted into the 8F Cerebase. The microcatheter was advanced over the microwire into the left distal ICA. A 5.0mm x 20mm PED was deployed across the neck of the clinoidal aneurysm (Figure 3A-3B). Post-deployment left ICA angiogram demonstrated proper placement of the PED with evidence of contrast stasis within the left clinoidal aneurysm (Figure 4A-4B). The patient was closely monitored in the Neurocritical Care Unit overnight and was discharged home in post-procedure day two with reported improvement in her headaches and neurologically intact condition. 

## Discussion

The case we present details a complication with device deployment that is likely related to a combination of incomplete opening of the flow diverter and foreshortening of the device after deployment. We postulate that the tortuosity in the proximal segment of the cavernous ICA and superimposed mild parent vessel stenosis in the proximal cavernous ICA provided the environment for incomplete opening of the flow diverting device. Additionally, the foreshortening of the device resulted in inadequate coverage of the aneurysm’s neck. In an attempt to provide adequate coverage of the aneurysm’s neck, the decision was made to place another FRED device distally. However, upon attempting to navigate the microwire through the FRED, we were unable to obtain distal access due to proximal distortion of the FRED. Successful retrieval of the flow diverter was performed with the utilization of a microsnare without injury to the parent vessel or aneurysmal rupture. This complication related to this flow diverter has not been reported in the literature previously and has only been reported once with the Pipeline Embolization Device (PED; Medtronic, Irvine, California, USA), making it an exceedingly rare complication with potentially disastrous thromboembolic complications. There is insufficient experience, and therefore evidence or commentary, to discuss the benefits of the distal and proximal radiopaque markers of FRED, as well as the greater fluoroscopic visibility of the tantalum coated interwoven wires of the stent, versus the potential difficulty in deployment and risk of developing this complication. 

Retrieval of retained endovascular devices has been successfully reported in the literature by the use of stent retrievers and microsnares for retained low profile intracranial stents, stent retrievers, flow diverting stents, microcatheters, coils, and the Woven Endobridge device (WEB; Microvention, Aliso Viejo, California, USA) [4-10]. Mitchell et al. successfully demonstrated microsnare retrieval of a distorted Pipeline Embolization Device (PED; Medtronic, Irvine, California, USA). Furthermore, the authors describe the indication for continued treatment of the aneurysm, after microsnare retrieval of the initial device, was to prevent aneurysmal rupture and to treat any parent vessel dissection that may have occurred with microsnare retrieval of the initial flow diverting device [8]. We were able to successfully retrieve the FRED in our patient using a microsnare and placed a PED without any complications. Our case can be used as a reference in the future for the successful management of this complication. 

## Conclusions

We report, to our knowledge, the first case of microsnare retrieval of a distorted FRED. This case serves to document this complication with this flow diverting device, as well as to further document the utility of microsnares in the retrieval of endovascular devices. Incomplete opening of the FRED should prompt immediate treatment to decrease the risk of thromboembolic complications.

## Appendices

1. EuFRED: European Multicenter Study for the Evaluation of a Dual-Layer Flow-Diverting Stent for Treatment of Wide-Neck Intracranial Aneurysms: The European Flow-Redirection Intraluminal Device Study

2. SAFE: Safety and Efficacy Analysis of FRED Embolic Device in Aneurysm treatment.  
